# Supplementary material for: Bayesian refinement of protein structures and ensembles against SAXS data using molecular dynamics
Source: PLoS Comput Biol. 2017 Oct 18;13(10):e1005800. doi: 10.1371/journal.pcbi.1005800 (PMC5662244; doi:10.1371/journal.pcbi.1005800)
Supplement: S1 Archive — (BZ2) [file pcbi.1005800.s011.tar.bz2 › gromacs_4.62_bayesian_saxs_refi/share/html/online/g_pme_error.html]

g\_pme\_error


| g\_pme\_error Main Table of Contents |

**VERSION 4.6  
Sat 19 Jan 2013**

---

### Description

g\_pme\_error estimates the error of the electrostatic forces
if using the sPME algorithm. The flag -tune will determine
the splitting parameter such that the error is equally
distributed over the real and reciprocal space part.
The part of the error that stems from self interaction of the particles is computationally demanding. However, a good a approximation is to
just use a fraction of the particles for this term which can be
indicated by the flag -self.

### Files

| option | filename | type | description |
| --- | --- | --- | --- |
| **-s** | topol.tpr | Input | Run input file: tpr tpb tpa |
| **-o** | error.out | Output | Generic output file |
| **-so** | tuned.tpr | Output, Opt. | Run input file: tpr tpb tpa |

### Other options

| option | type | default | description |
| --- | --- | --- | --- |
| **-[no]h** | bool | no | Print help info and quit || **-[no]version** | bool | no | Print version info and quit || **-nice** | int | 0 | Set the nicelevel || **-beta** | real | -1 | If positive, overwrite ewald\_beta from .tpr file with this value || **-[no]tune** | bool | no | Tune the splitting parameter such that the error is equally distributed between real and reciprocal space || **-self** | real | 1 | If between 0.0 and 1.0, determine self interaction error from just this fraction of the charged particles || **-seed** | int | 0 | Random number seed used for Monte Carlo algorithm when -self is set to a value between 0.0 and 1.0 || **-[no]v** | bool | no | Be loud and noisy |

---

http://www.gromacs.org  
gromacs@gromacs.org
